# Interleukin-6-derived cancer-associated fibroblasts activate STAT3 pathway contributing to gemcitabine resistance in cholangiocarcinoma

**DOI:** 10.3389/fphar.2022.897368

**Published:** 2022-08-26

**Authors:** Yingpinyapat Kittirat, Manida Suksawat, Suyanee Thongchot, Sureerat Padthaisong, Jutarop Phetcharaburanin, Arporn Wangwiwatsin, Poramate Klanrit, Sakkarn Sangkhamanon, Attapol Titapun, Watcharin Loilome, Hideyuki Saya, Nisana Namwat

**Affiliations:** ^1^ Department of Biochemistry, Faculty of Medicine, Khon Kaen University, Khon Kaen, Thailand; ^2^ Cholangiocarcinoma Research Institute, Faculty of Medicine, Khon Kaen University, Khon Kaen, Thailand; ^3^ Khon Kaen University International Phenome Laboratory, Khon Kaen University Science Park, Innovation and Enterprise Affairs, Khon Kaen University, Khon Kaen, Thailand; ^4^ Department of Immunology, Faculty of Medicine Siriraj Hospital, Mahidol University, Bangkok, Thailand; ^5^ Siriraj Center of Research Excellence for Cancer Immunotherapy (SiCORE-CIT), Research Department, Faculty of Medicine Siriraj Hospital, Mahidol University, Bangkok, Thailand; ^6^ Faculty of Allied Health Sciences, Burapha University, Chonburi, Thailand; ^7^ Department of Pathology, Faculty of Medicine, Khon Kaen University, Khon Kaen, Thailand; ^8^ Department of Surgery, Faculty of Medicine, Khon Kaen University, Khon Kaen, Thailand; ^9^ Division of Gene Regulation, Fujita Cancer Center, Fujita Health University, Tokyo, Japan

**Keywords:** cholangiocarcinoma, IL-6, gemcitabine, cancer-associated fibroblasts, tocilizumab

## Abstract

Cancer-associated fibroblasts (CAFs) are the dominant component of the tumor microenvironment (TME) that can be beneficial to the generation and progression of cancer cells leading to chemotherapeutic failure *via* several mechanisms. Nevertheless, the roles of CAFs on anti-cancer drug response need more empirical evidence in cholangiocarcinoma (CCA). Herein, we examined the oncogenic roles of CAFs on gemcitabine resistance in CCA cells mediated *via* IL-6/STAT3 activation. Our findings showed that CCA-derived CAFs promote cell viability and enhance gemcitabine resistance in CCA cells through the activation of IL-6/STAT3 signaling. High expression of IL-6R was correlated with a poor overall survival rate and gemcitabine resistance in CCA, indicating that IL-6R can be a prognostic or predictive biomarker for the chemotherapeutic response of CCA patients. Blockade of IL-6R on CCA cells by tocilizumab, an IL-6R humanized antihuman monoclonal antibody, contributed to inhibition of the CAF-CCA interaction leading to enhancement of gemcitabine sensitivity in CCA cells. The results of this study should be helpful for modifying therapeutic regimens aimed at targeting CAF interacting with cancer cells resulting in the suppression of the tumor progression but enhancement of drug sensitivity.

## Introduction

Cholangiocarcinoma (CCA) is a bile duct cancer originating from cholangiocytes lining of biliary tract. CCA is the second most common primary liver cancer worldwide (Banales et al., 2016). This cancer occurs prevalently in mainland Southeast Asian countries, especially in Northeast Thailand which has the world’s highest incidence rates (Sriplung et al., 2006; Hughes et al., 2017). A poor prognosis and low survival rate are burdens for CCA treatment (Khan et al., 2012; Khuntikeo et al., 2014). Surgical resection has been the mainstay remedy in CCA treatment. CCA patients with unresectable cancer often received chemotherapy as a palliative treatment (Thongprasert, 2005; Butthongkomvong et al., 2013). However, CCA is resistant to common chemotherapy and has a poor response based on the characteristics of its multidrug resistant phenotypes, leading to complex mechanisms of chemoresistance (Marin et al., 2018). The lack of sensitivity of CCA cells to chemotherapeutic drugs is still dismal. The mechanisms of chemoresistance are composed of drug metabolism (such as its uptake, export, and intracellular biotransformation), the expression of molecular targets on cancer cells, and the ability of repair mechanisms to avoid apoptosis or programmed cell death (Banales et al., 2016). Moreover, the extensive desmoplastic microenvironment surrounding the neoplastic ducts, termed tumor reactive stroma, act as a key determinant of decreased sensitivity of CCA to drug-induced cytotoxicity (Cadamuro et al., 2017). To improve the potential of anti-cancer drugs for CCA treatment we should consider their mechanisms of chemoresistance.

The etiology of Thai CCA is strongly associated with chronic infection by the liver fluke (*Opisthorchis viverrini*), resulting in chronic inflammation that contributes to biliary damage (Sripa and Pairojkul, 2008; Songserm et al., 2009). Repeated infection causes tissue injury followed by chronic wound healing of host repair resulting in the generation of cancerous lesions that contains heterotypic tumor and stromal cells. The tumor microenvironment (TME) or tumor stroma consists of heterogeneous stromal cell populations. A complex network of extracellular matrix (ECM) surrounding cancer cells is beneficial to cancer development and progression (Pietras and Ostman, 2010; Quail and Joyce, 2013). A dominant component of TME is activated fibroblasts that are termed cancer-associated fibroblasts (CAFs). Several previous studies suggested functional roles for these cells in cancer progression and drug resistance mediated by secretory molecules such as cytokines or chemokines and physical contact *via* cell-cell adhesion molecules resulting in cancer cell growth and ECM remodeling toward migration and invasive outgrowth (Kalluri and Zeisberg, 2006; Ohlund et al., 2014). The alteration of pathways associated with the interaction of CAFs and cancer cells, including ECM adhesion and paracrine signaling, also facilitates mechanisms for cancer resistance (Meads et al., 2009; Paraiso and Smalley, 2013). Hence, the tumor stroma is the major causes of cancer development and progression toward chemoresistance presenting a clinical challenge. (Hogdall et al., 2018). Moreover, CAFs promote a migration of CCA cells *via* interleukin-6 (IL-6) secretion driving epithelial-to-mesenchymal transition (Thongchot et al., 2018). High expression of IL-6 in fibrotic stromal cells is associated with a poor prognosis and chemoresponse in CCA patients (Thongchot et al., 2021). The inhibitor related to the blockage of IL-6 and IL-6R such as tocilizumab is considered as an effective anti-cancer therapeutic approach (Masjedi et al., 2018). Therefore, improved understanding of interaction between CCA cells and their microenvironment might be effective strategies to overcome limitations on CCA treatment.

The development of drug resistance and CAFs are influential in promoting cancer cell evasion of anti-cancer therapies. However, the mechanisms involved in drug resistance are still poorly defined. Taking all data together, they suggest that CAFs play important roles in CCA progression and drug resistance. Understanding the mechanism by which CAFs communicate or interact with CCA cells is likely to be useful in the discovery of predictors of chemotherapeutic response toward drug targeting. In this study, we aimed at investigating the roles of CCA-derived CAFs on gemcitabine resistance in CCA cells and the inhibition of CAF-CCA interactions by blocking IL-6R on CCA cells to enhance gemcitabine sensitivity. These findings will be helpful for unraveling more secrets of CAFs on their pro-oncogenic effects in CCA chemoresistance, as well as modifying the therapeutic regimen aimed at targeting their interaction resulting in suppression of the tumor progression and enhancement of drug sensitivity.

## Materials and methods

### Patients, samples, and ethical issues

CCA tissue microarray (CCA-TMAs) of paraffin-embedded cases originated from primary tumors of 146 patients who admitted to surgical wards of Srinagarind Hospital, Khon Kaen University, Khon Kaen, Thailand, collected between 2014–2016. CAF samples were also collected from CCA tissues of patients who underwent surgical resection in Srinagarind Hospital. Written informed consent was obtained from all patients in accordance with the Declaration of Helsinki and its later revision. The Human Research Ethics Committee, Khon Kaen University, approved the research protocol (#HE571283 and #HE611544).

### Reagents

MTT (3-(4,5-dimethylthiazol-2-yl)-2,5-diphenyltetrazolium bromide) formazan and dimethyl sulfoxide (DMSO) were purchased from Sigma-Aldrich (St. Louis, MO). Human IL-6 Quantikine ELISA Kit was purchased from R&D system (Minnesota, United States). Recombinant IL-6 was purchased from ImmunoTools (Friesoythe; DE), Gemcitabine was purchased from Fresenius Kabi (Maharashtra, IN), IL-6R inhibitor (Tocilizumab) was purchased from Roche (Basel, CH).

### Cell culture

CCA cell lines, KKU-055 and KKU-213A, were obtained from the Japanese Collection of Research Bioresources Cell Bank (Osaka, Japan). The KKU-055 and KKU-213A cell lines were isolated from the tissue of 56- and 58-year-old male CCA patients, respectively. Both cell lines are established and characterized as poorly differentiated cells (Sripa et al., 2020). Primary normal adult human dermal fibroblast (NF) (ATCC® PCS-201–012™) was purchased from American Type Culture Collection (ATCC) (Virginia, US). Cells were cultured in Dulbecco’s Modified Eagle Medium (DMEM) supplemented with 10% heat-inactivated fetal bovine serum (FBS) and 100 IU/ml of penicillin-streptomycin, at 37°C in a humidified atmosphere containing 5% CO_2_.

### Primary cultured cancer-associated fibroblasts isolation and culture

The samples from 8 CCA patient’s tissues were cut into small pieces, digested with 0.5% collagenase, and filtered through the cell strainer. The isolated cells were cultured in DMEM containing 10% fetal bovine serum with 100 U/ml penicillin, 100 μg/ml streptomycin. CAFs were identified by morphology and staining for a CAF marker, α-smooth muscle actin (α-SMA). The patients’ clinical information was presented in Supplementary Table S1.

### Immunofluorescence assay

Primary CAF culture (6 × 10^4^ cells/ml) were plated in a slide chamber and incubated at 37°C in a humidified 5% CO_2_ atmosphere. After 24 h incubation, CAFs were fixed by 4% paraformaldehyde and blocked with 5% bovine serum albumin (BSA) followed by incubation with primary specific antibodies including anti-α-SMA (Abcam, Cambridge, United Kingdom) and anti-cytokeratin-19 (CK-19) (Abcam, Cambridge, United Kingdom). After incubation, the secondary antibody Alexa Fluor® 488 and 555 (Invitrogen, Massachusetts, United States) were added into cells. The slides were mounted with 50% glycerol in phosphate buffered saline (PBS) contained Hoechst 33342 (Invitrogen, Massachusetts, United States) nucleic acid stain.

### Transwell co-culture system

Non-contact co-culture of CCA cells (KKU-055 and KKU-213A) and primary isolated CAFs was performed using a Transwell assay by plating CAFs into the 6.5 mm insert while plating CCA cells in the bottom of 24-well plates. After 16 h incubation, the inserts containing CAFs were placed on the CCA cells. A DMEM containing 1% FBS was used in this experiment. Cell viability of CCA cells in a co-culture plate was measured using MTT assay.

### MTT assay

After experiments, the cell viability was determined by adding the 0.5 mg/ml of MTT solution into each well and incubated at 37°C for 1 h. After incubation, dimethyl sulfoxide (DMSO) was added to dissolve formazan. The absorbance was determined at 540 nm using a microplate reader (Tecan, Männedorf, CH).

### Gemcitabine and IL-6R inhibitor treatment in the co-culture

After co-culturing CAFs and CCA cells for 72 h, gemcitabine was added into the co-culture at a final concentration based on the half maximal inhibitory concentration (IC_50_) of CCA cells for 48 h. Additionally, for the IL-6R inhibitor treatment, CCA cells were pre-treated with the IL-6R inhibitor for 6 h followed by co-culturing with CAFs and gemcitabine treatment. Cell viability of CCA cells was measured using an MTT assay after 48-h post-treatment.

### Enzyme-linked immunosorbent assay

Conditioned media (CM) from CAFs and the co-cultures were collected after incubation with and without gemcitabine. CM were centrifuged to remove the debris and the ELISA was immediately performed following the manufacturer’s protocol for the human IL-6 ELISA kit (R&D system).

### Western blot analysis

CCA cells were harvested and then lysed with RIPA lysis buffer (150 mM NaCl, 0.5 M Tris-HCl pH 7.4, 1% (v/v) Tween-20, 1% (w/v) sodium deoxycholate, 0.1% (w/v) SDS). Protein concentration was determined using the Pierce BCA^TM^ Protein Assay kit (Pierce Biotechnology, Rockford, United States). The 20 μg of protein extracts were electrophoresed by 10% (w/v) sodium dodecyl sulfate polyacrylamide gel electrophoresis (SDS-PAGE), transferred to a PVDF membrane, and blocked with 5% (w/v) skimmed milk. The membranes were probed at 4°C overnight with the following antibodies: rabbit anti-pSTAT3 (Abcam, Cambridge, United Kingdom), rabbit anti-STAT3 (Cell Signaling Technology, Massachusetts, US), mouse anti-IL-6R (OriGene, Maryland, US), rabbit anti-Bcl-2 (Proteintech, Illinois, US) and rabbit anti-Cyclin D1 (Cell Signaling Technology, Massachusetts, US) antibodies and β-actin antibody (Proteintech, Illinois, US) was used as an internal loading control. After incubation with secondary antibodies the band intensity was measured using ECL^TM^ Prime Western Blotting Detection Reagent for chemiluminescent detection (GE Healthcare, Illinois, US). The apparent density of the bands on the membranes was captured by Image Quant Imager (GE Healthcare, Illinois, US).

### Immunohistochemical analysis

IL-6R was detected on the CCA tissue microarray (TMA) of 146 paraffin embedded sections using standard immunohistochemistry protocols. Firstly, tissue sections were de‐paraffinized and rehydrated with stepwise xylene followed by a series of concentrations of ethanol. Antigen retrieval was performed by microwaving sections in 10 mM sodium citrate pH 6 for 10 min. After that, the tissue sections were treated with 0.3% H_2_O_2_ for 30 min to block endogenous peroxidase activity. Nonspecific binding was blocked by 10% skim milk in phosphate‐buffered saline (PBS) for 1 h. Sections were incubated with primary antibody (IL6R Antibody #CF506859; Thermo Fisher, Massachusetts, United States) for 1 h at room temperature followed by 4°C overnight. Thereafter, the sections were washed in PBS containing 0.1% tween 20 and incubated with peroxidase‐conjugated Envision secondary antibody (DAKO, Glostrup, Denmark) for 1 h. The color was developed with a 3,3′diaminobenzidine tetrahydrochloride (DAB) substrate kit (Vector Laboratories, Inc., CA) for 5 min and the sections were counterstained with Mayer’s hematoxylin for 2 min and dehydrated stepwise with a series of concentrations of ethanol and xylene. Finally, sections were mounted with permount and observed under a light microscope (Eclipse, Ni‐U, Nikon Instru‐ments Inc. Missouri, United States). The protein expression was analyzed according to staining frequency and intensity. The staining frequency of proteins was semiquantitatively scored based on the percentages of positive cells, 0% = negative; 1–25% = +1; 26–50% = +2; and >50% = +3. The intensity of protein staining was scored as weak = 1, moderate = 2, and strong = 3. The final immunohistochemical score was determined by multiplying the intensity with the frequency score. The median value was calculated by grading the scores of all patients and was used as a cut-off point. The patients with grading score lower than the median were classified as the low expression group and those with a grading score equal to or higher than the median was classified as the high expression group (Namwat et al., 2011).

### Statistical analysis

The data were collected from at least 3 independent experiments and processed using GraphPad Prism 5.0 software (GraphPad. Software Inc.). The statistical analysis was performed using ANOVA and Student’s t-test. Immunohistochemical analyses were carried out using Statistical Package for the Social Sciences; SPSS software (version 27.0; SPSS Inc., Chicago, IL, United States). A survival curve was calculated using the Kaplan-Meier (log-rank) analysis. The analyses of factors predicting overall survival was determined using Cox proportion hazard regression. The correlation between IL-6R expression score and HDRA data was performed using a Pearson’s correlation. A *p*-value of less than 0.05 was considered statistically significant.

## Results

### Isolation and characterization of cancer-associated fibroblasts

**FIGURE 1:**
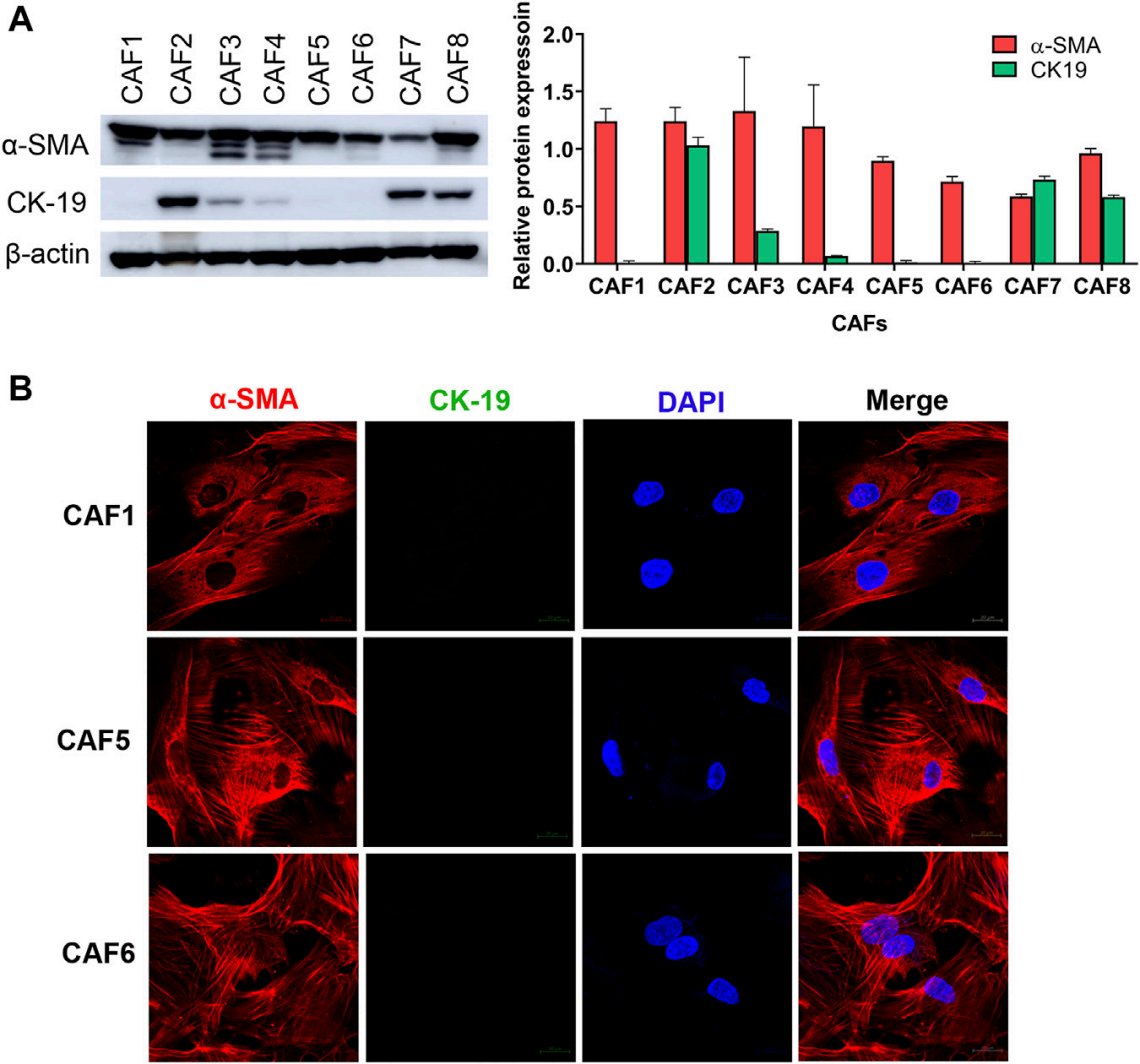
Characterization of CAFs by western blotting and immunofluorescence staining. **(A)** Western blot analysis shows the levels of α-SMA and CK-19 in primarily isolated CAFs. **(B)** CAFs displayed a spindle-like morphology by α-SMA staining observed under confocal microscopy. Hoechst 33342 was used for visualizing the nuclei of CAFs. Green, stained with cytokeratin-19 (CK-19); red, stained with α-SMA and blue, stained with Hoechst 33342. Original magnification is ×630.

To investigate the effect of CAFs on the gemcitabine response of CCA cells, we isolated CAFs from 8 CCA patients’ tissues. Since the isolated CAFs present a spindle-like morphology, we performed western blotting to examine the expression level of a CAF marker, i.e., α-SMA. CK-19, an epithelial cell marker, was used to confirm non-contamination with epithelial cells. The results showed that all isolated CAFs expressed α-SMA. The 3 isolated CAFs showed no expression of CK-19 indicating that these CAFs were not contaminated with epithelial cells including CAF1, CAF5 and CAF6 (Figure 1A). Moreover, these 3 CAFs were further verified by immunofluorescence staining. In Figure 1B, CAFs showed positive staining with α-SMA presenting as a red color, but they were not stained with CK-19 (green). In sub-culturing passages, CAF1 stopped dividing and it could not be subsequently cultured. Therefore, the 2 isolated CAFs (CAF5 and CAF6) were collected for experiments.

### IL-6 induced gemcitabine resistance of cholangiocarcinoma cells

**FIGURE 2:**
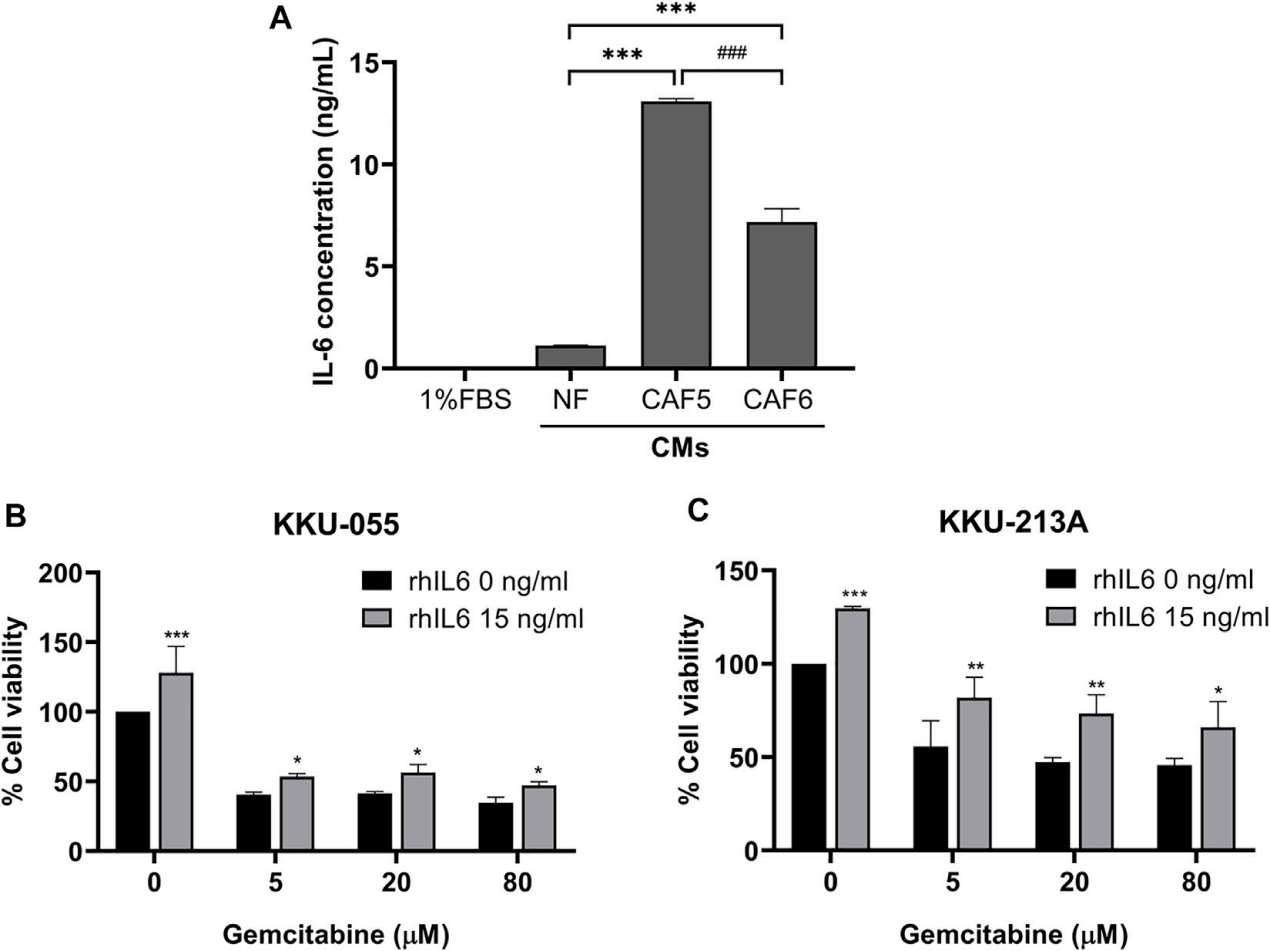
The level of IL-6 secreted from NF, CAFs and efficacy of rhIL-6 on gemcitabine response of CCA cells. **(A)** A human IL-6 ELISA kit was used for analyzing the level of IL-6 in conditioned medium (CMs) from NF and CAFs. **(B,C)** Cell viability was measured in CCA cells (KKU-055 and KKU-213A) by MTT assay upon treatment with rhIL-6 and gemcitabine. Error bars represent the standard deviation (SD) of triplicate experiments. A significant difference was determined using ANOVA and unpaired t-test (*p < 0.05, **p < 0.01, ***p < 0.001 compared to the control groups, ^###^ *p* < 0.001 compared between CAF5 and CAF6).

We determined the level of IL-6 secreted from NF and CAFs using a human IL-6 ELISA kit. The NF was used as a control for determining the basal level of IL-6 in normal fibroblasts. The 1% (v/v) FBS-conditioned media was used as a reference value for comparison. The result showed that the level of IL-6 in CAFs was significantly higher than NF. It was noted that the IL-6 level in CAF5 was significantly higher than that in CAF6 (Figure 2A). Therefore, CAF5 was selected to perform further experiments. In addition, rhIL-6 was used for examining the effect of IL-6 on the gemcitabine response of CCA cells. CCA cells were pre-treated with 15 ng/ml of rhIL-6 for 72 h and then treated with gemcitabine at 5, 20 and 80 μM for 48 h. The half-maximal inhibitory concentrations (IC_50_) at 48 h of gemcitabine on CCA cells including KKU-055 and KKU213A were 3.50 ± 1.0 and 10.1 ± 14.0 μM, respectively. The IC_50_ of gemcitabine on KKU-055 and KKU-213A cells in presence of rhIL-6 were 54.4 ± 18.0 and 705.1 ± 18.6 μM, respectively. The result showed that rhIL-6 treated CCA cells had a significant higher IC_50_ and percentage of cell viability than untreated cells upon gemcitabine treatment (Figures 2B,C).

### Cholangiocarcinoma-derived cancer-associated fibroblasts induced gemcitabine resistance of cholangiocarcinoma cells

**FIGURE 3:**
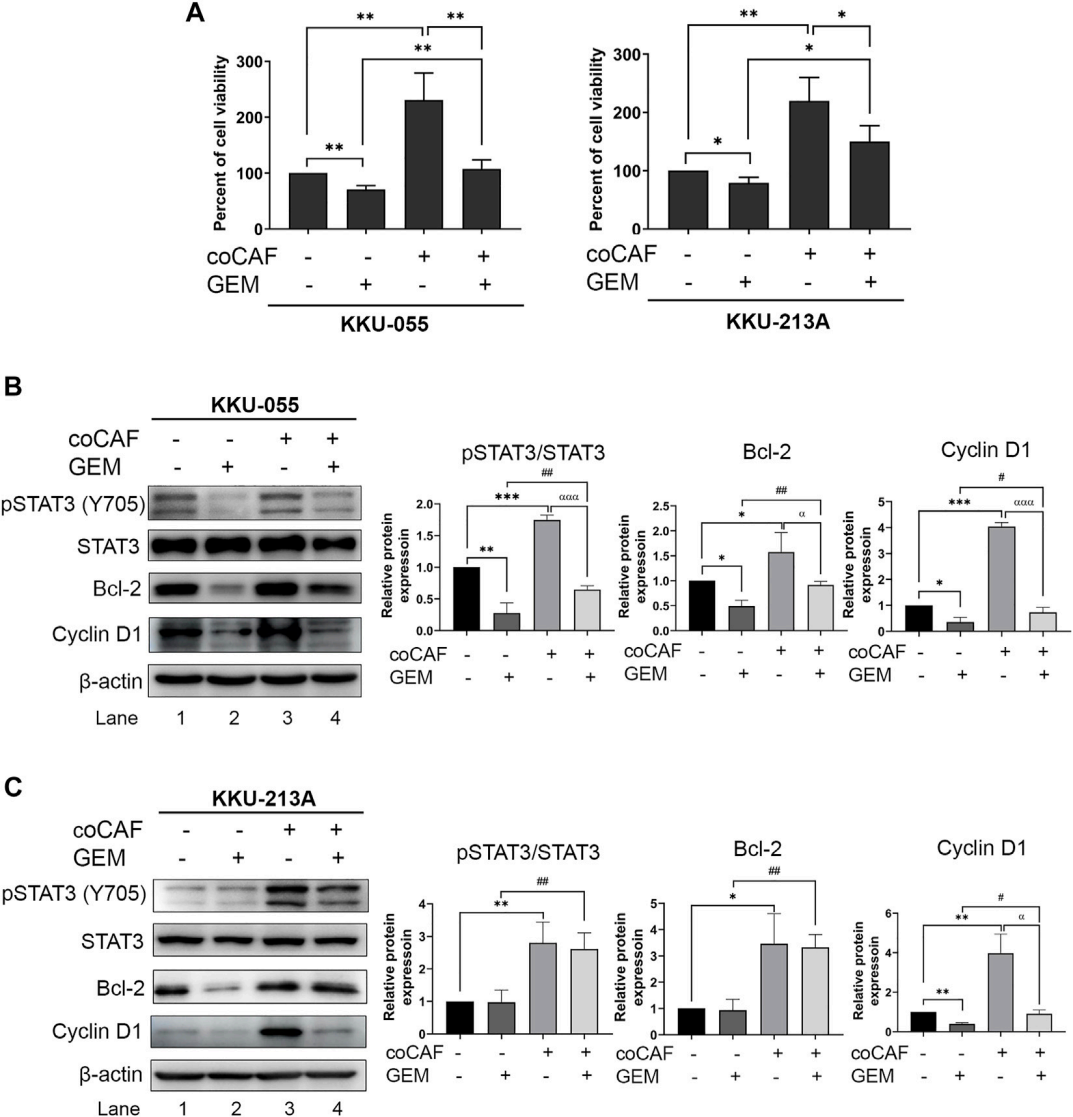
The effects of CAFs and molecular changes on gemcitabine response of CCA cells. **(A)** CCA cell viability after incubation with and without CAFs and gemcitabine were determined by MTT assay. Western blotting shows the molecular mechanisms of **(B)** KKU-055 and **(C)** KKU-213A assessed after monoculture or co-culture with CAFs and treatment with or without gemcitabine. Graphical plots represent the relative protein expressions. Error bars represent the standard deviation (SD) of triplicate experiments. A significant difference was determined using ANOVA and unpaired t-test (*p < 0.05, **p < 0.01, ***p < 0.001) compared to those control groups (black bars), ^#^ *p* < 0.05, ^##^ *p* < 0.01 compared to those gemcitabine-treated groups (dark grey bars), ^α^ *p* < 0.05, ^ααα^ *p* <0.001 compared to those co-cultures (grey bars).

We investigated the effect of CCA-derived CAFs on gemcitabine response in CCA cells. CCA cells were co-cultured with CAF5 cells for 72 h. After incubation, the co-cultures were treated with gemcitabine for 48 h and cell viability measured by MTT assay. We found that the co-cultures significantly increased their viability up to 1.5- and 1.9-fold in KKU-055 and KKU-213A, respectively when compared to the monoculture. The co-cultures treated gemcitabine significantly increased cell viability in KKU-055 and KKU-213A up to 0.5- and 0.8-fold, respectively when compared to monoculture treated with gemcitabine (Figure 3A), indicating that CAFs induced CCA cells to be resistant against gemcitabine. The molecular changes were also investigated in gemcitabine treated CCA cells after incubation with or without CAFs using western blot analysis. The results showed that co-cultures treated with gemcitabine significantly activated the STAT3 phosphorylation level and induced the expression of Bcl-2 and Cyclin D1 compared to the treated monoculture groups in KKU-055 (Figure 3B, lane 2 and 4) and KKU-213A (Figure 3C, lane 2 and 4). The results imply that CAFs interact and promote gemcitabine resistance mediated by the STAT3 signaling in CCA cells.

### IL-6R inhibitor suppressed pSTAT3 level in rhIL-6-treated cholangiocarcinoma cells

**FIGURE 4:**
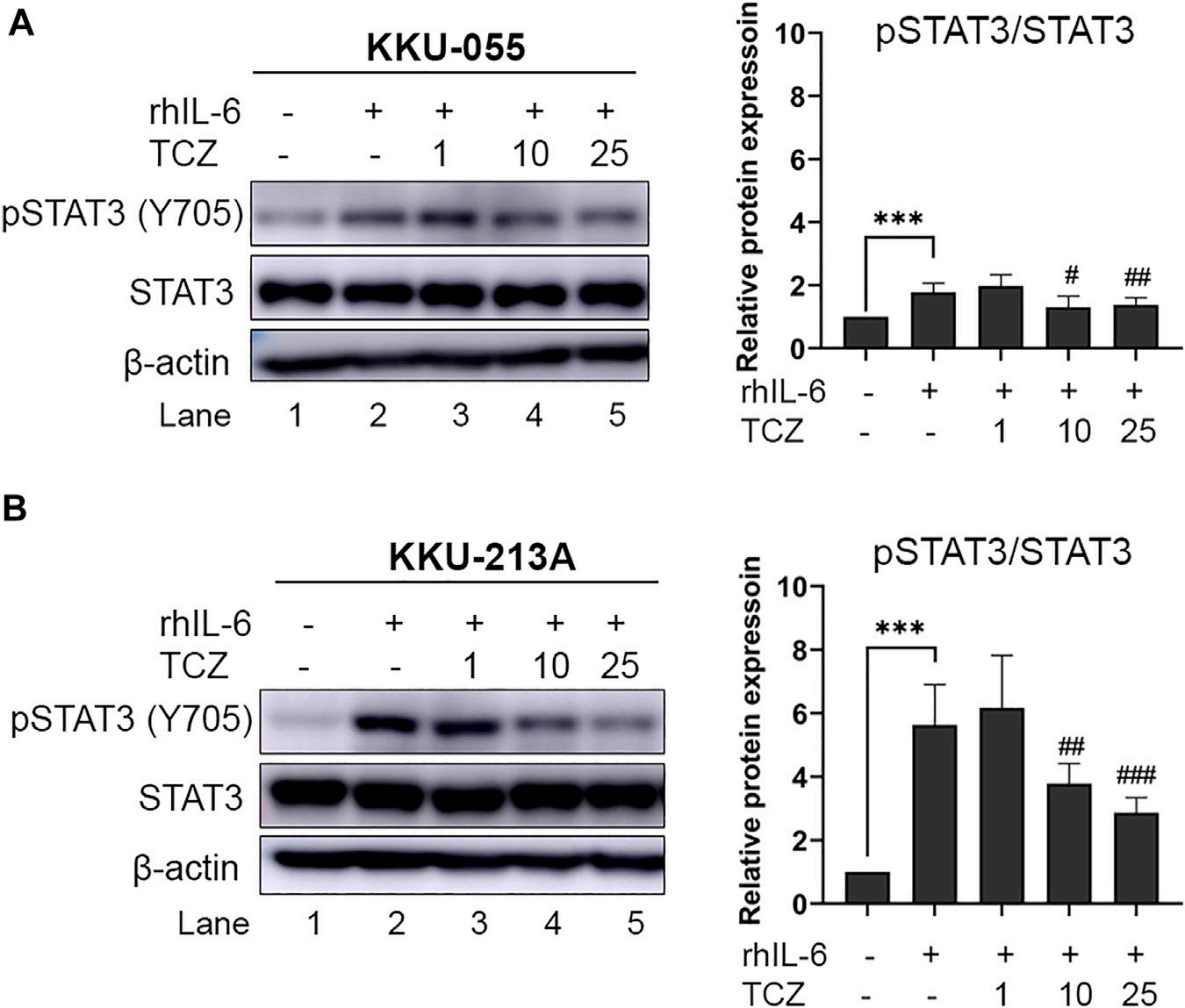
The efficacy of IL-6R inhibitor (Tocilizumab) on pSTAT3 levels of rhIL-6-treated CCA cells. The level of pSTAT3 in **(A)** KKU-055 and **(B)** KKU-213A after treatment with different concentrations of TCZ was assessed by western blot analysis. The graphical plots demonstrate relative protein expression of pSTAT3 and total STAT3. TCZ; tocilizumab. Error bars represent the standard deviation (SD) of triplicate experiments. A significant difference was determined using ANOVA and unpaired t-test (***p < 0.001 compared to untreated groups, ^#^ *p* < 0.05, ^##^ *p* < 0.01, ^###^ *p* < 0.001 compared to rhIL-6 treated groups).

To disrupt the interaction between CCA cells and CAFs *via* IL-6 signal transduction the IL-6R inhibitor (Tocilizumab; TCZ) was used. CCA cells were pre-incubated with rhIL-6 to induce pSTAT3 levels. After incubation, TCZ (1, 10 and 25 μg/ml) was added into the CCA cell culture for 6 h. The results showed that TCZ at concentrations of 10 and 25 μg/ml significantly suppressed pSTAT3 levels in rhIL-6-treated KKU-055 cells (Figure 4A, lane 4 and 5) and KKU-213A (Figure 4B, lane 4 and 5), indicating that TCZ inhibited the interaction of CAFs and CCA cells mediated by IL-6/STAT3 axis.

### Blocking IL-6/STAT3 axis suppressed IL-6 secretion from cancer-associated fibroblasts-cholangiocarcinoma interaction and enhanced gemcitabine sensitivity in cholangiocarcinoma cells

**FIGURE 5:**
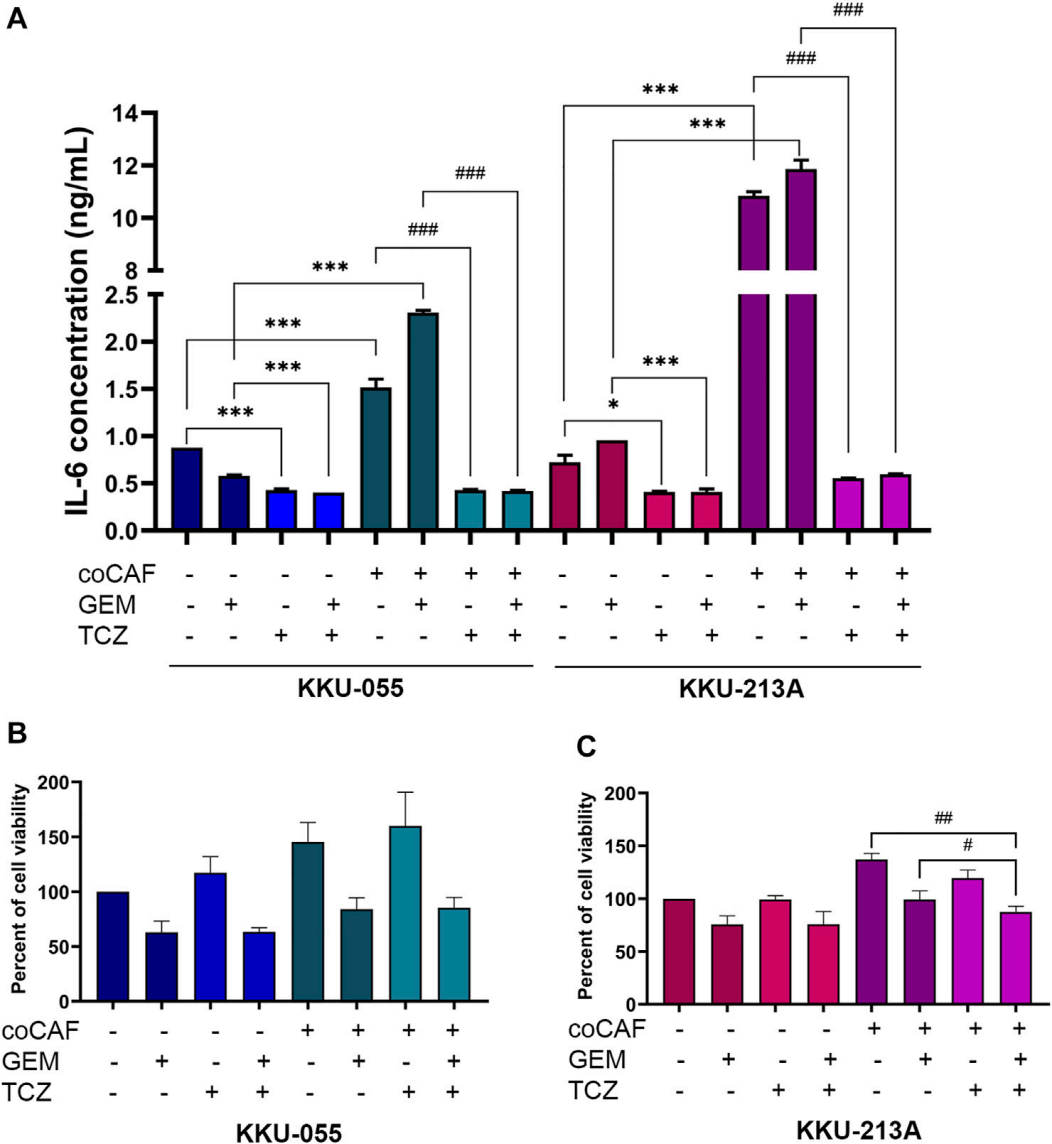
Effect of IL-6R inhibitor (TCZ) on gemcitabine response of CCA cells. **(A)** IL-6 levels in CMs from KKU-055 and KKU-213A in both mono- and co-culture were determined by human IL-6 ELISA kit. **(B,C)** CCA cell viability in culture with or without CAFs and after (or not after) gemcitabine and TCZ treatment was determined by MTT assay. TCZ; Tocilizumab. Error bars represent the standard deviation (SD) of triplicate experiments. A significant difference was determined using ANOVA and unpaired t-test (*p < 0.05, ***p < 0.001 compared to those monocultured groups, ^#^ *p* < 0.05, ^##^ *p* < 0.01, ^###^ *p* < 0.001 compared to co-cultured groups).

To investigate the effect of TCZ on IL-6 secretion from the CAF-CCA interaction, we pre-treated TCZ to suppress the IL-6 signaling through IL-6R on CCA cells. CCA cells were cultured with and without adding CAFs followed by gemcitabine treatment. The levels of IL-6 in conditioned media of CCA cells in monoculture and co-culture were measured using ELISA. The results showed that the IL-6 level in CCA cells under co-culture was significantly higher than those under monoculture of both cells. Besides, TCZ significantly suppressed IL-6 levels in the monoculture and co-culture in both CCA cells when compared to the untreated groups (Figure 5A). In Figures 5B,C, TCZ did not affect the viability in either CCA cell line. TCZ-treated co-cultured cells significantly reduced cell viability in gemcitabine treatment. These results indicated that blocking the CAF-CCA interaction *via* the IL-6/STAT3 axis enhanced gemcitabine sensitivity in KKU-213A and KKU-055.

**FIGURE 6:**
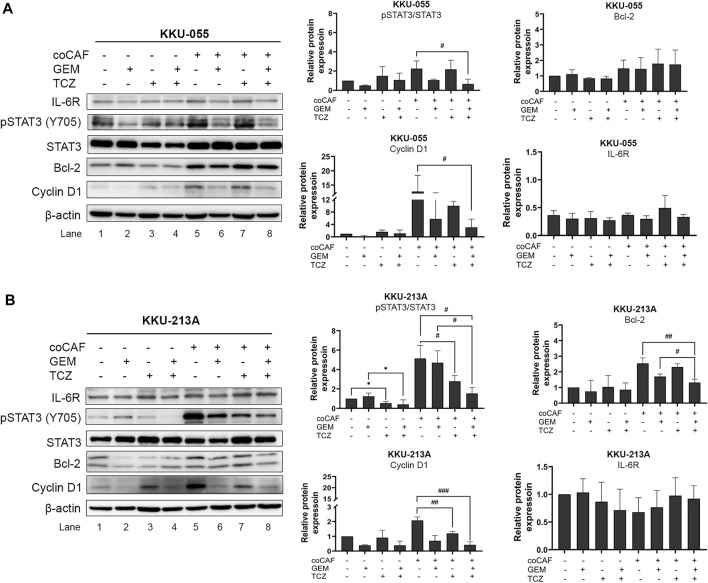
Effect of IL-6R inhibitor (TCZ) on molecular changes of CCA cells. The molecular mechanisms of **(A)** KKU-055 and **(B)** KKU-213A after co-culture with and without CAFs and then, incubation with and without gemcitabine and TCZ were assessed by western blotting. Error bars represent the standard deviation (SD) of triplicate experiments. A significant difference was determined using ANOVA and unpaired t-test (*p < 0.05 compared to those monocultured groups, ^#^ *p* < 0.05, ^##^ *p* < 0.01, ^###^ *p* < 0.001 compared to co-cultured groups).

The molecular mechanisms of TCZ on the inhibition of the CAF-CCA interaction mediated by IL-6/STAT3 toward the gemcitabine response of CCA cells were demonstrated by western blotting. After co-culture and treatment, CCA cells were collected to evaluate the molecular changes. In KKU-213A cells, TCZ significantly suppressed the pSTAT3 level when compared to the untreated co-culture (Figure 6B, lane 5 and 7). Likewise, a combination of TCZ and gemcitabine significantly decreased pSTAT3 level and cyclin D1 when compared to the untreated and TCZ treated-co-culture groups (Figure 6B, lane 5 and 8). This result indicates that TCZ can attenuate CAF-induced KKU-213A gemcitabine resistance through the inhibition of IL-6/STAT3 signaling leading to gemcitabine sensitivity. In contrast, the TCZ treated-co-culture did not significantly affect signaling in KKU-055 cells (Figure 6A, lane 5 and 7). However, TCZ combined with gemcitabine significantly suppressed pSTAT3 level and cyclin D1 when compared to the untreated co-culture group, which is consistent with the result in Figure 5B. These results suggest that KKU-055 and KKU-213A have different response mechanisms. It was noted that the expression level of IL-6R in KKU-055 was lower than that of KKU-213A, suggesting a lower response of KKU-055 cells to gemcitabine and TCZ.

### The prognostic significance of IL-6R expression and clinicopathological features

**FIGURE 7:**
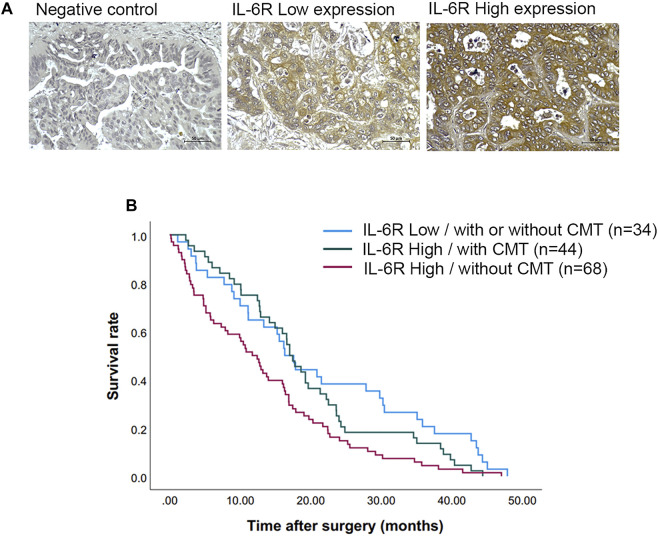
The expression of IL-6R in CCA tissues and correlation with overall survival rates of CCA patients. **(A)** Representative images demonstrated the expression of IL-6R by immunohistochemical staining in CCA tissues. Original magnification is ×200. **(B)** The Kaplan-Meier survival plot according to IL-6R expression and the chemotherapy regimen of CCA patients showed that the patients with IL-6R high expression without chemotherapy have a shorter survival time than those with low IL-6R expression and high IL-6R expression with chemotherapy (log-rank test, *p* = 0.005). CMT, chemotherapy.

Immunohistochemical (IHC) staining of IL-6R was performed in a CCA tissue microarray. The intensity of IHC staining were scored in the CCA area and ranged from negative (score = 0) to strong (score = 3). A total of 146 cases of CCA patients were studied with 56 (38%) female cases and 90 (62%) male cases. The ages ranged between 42 and 82 years (median = 61 years). Positive staining of IL-6R protein was seen in the cytoplasm and cell membrane in the tissue sections. To examine the association of IL-6R expression and the chemo-response in CCA patients, the patients were divided into those with IL-6R low expression, who were or were not subjected to any chemotherapy (34 cases), and those with IL-6R high expression who were subjected to chemotherapy (44 cases) and those with IL-6R high expression who were not subjected to chemotherapy (68 cases). The representative figures of negative control, IL-6R low and high expression in CCA are shown in Figure 7A. Log-rank analysis was carried out to determine the overall survival rate of patients with CCA according to the expression of IL-6R. The Kaplan-Meier plot demonstrated that a high expression of IL-6R in CCA patients who were not subjected to chemotherapy was significantly associated with a shortest overall survival when compared to other groups (*p* = 0.005) (Figure 7B). Additionally, the correlation between the expression level of IL-6R and clinicopathological features including sex, age, histological types, tumor staging, tumor site, lymph node metastasis and distant metastasis showed no significant correlation with IL-6R expression. All clinicopathological features were also analyzed by univariate Cox proportional hazards regression to identify prognostic factors for CCA patients. The results demonstrated that high expression of IL-6R without chemotherapy, high tumor staging, and intrahepatic site were significantly correlated with a shorter overall survival rate compared with those individuals with a low expression with chemotherapy, low tumor staging and extrahepatic site (*p* = 0.002, *p* = 0.034 and *p* = 0.010, respectively) (Table 1). In addition, multivariate analysis showed that a high expression of IL-6R and high tumor staging could be used as independent prognostic factors of clinicopathological characteristics for overall survival of CCA patients (*p* = 0.017; HR = 1.731; CI = 1.103–2.716 and *p* = 0.014; HR = 1.548; CI = 1.093–2.192, respectively) (Table 2).

**TABLE 1:** Univariate analysis of factors predicting overall survival of CCA patients.

Factors	No. of patients	Hazard ratio (HR)	95% Confidence Interval (CI)	*p*-value
IL-6R				
Low with/without CMT	34	1		
High with CMT	44	1.295	0.818–2.050	0.269
High without CMT	68	1.945	1.269–2.983	0.002*
Sex				
Female	56	1		
Male	90	0.946	0.673–1.330	0.752
Age				
<61	66	1		
≥61	80	1.174	0.845–1.631	0.338
Histological types				
Papillary	75	1		
Non-papillary	71	1.231	0.884–1.714	0.218
Tumor staging				
I/II	52	1		
III/IV	94	1.448	1.029–2.038	0.034*
Tumor site				
Intrahepatic	94	1		
Extrahepatic	52	0.632	0.445–0.898	0.010*
Lymph node metastasis				
No	129	1		
Yes	17	0.721	0.433–1.200	0.208
Distant metastasis				
No	103	1		
Yes	43	0.717	0.501–1.027	0.070

Univariate analysis by Cox proportion hazard regression. HR, indicates hazard ratio. 95% CI, refers to the 95% confidence interval. CMT, refers to chemotherapy. *p-values less than 0.05 were considered as a statistical significance.

**TABLE 2:** Multivariate analysis of factors predicting overall survival of CCA patients.

Factors	No. of patients	Hazard ratio (HR)	95% Confidence Interval (CI)	*p*-value
IL-6R				
Low with/without CMT	34	1		
High with CMT	44	1.157	0.724–1.849	0.541
High without CMT	68	1.731	1.103–2.716	0.017*
Tumor staging				
I/II	52	1		
III/IV	94	1.548	1.093–2.192	0.014*
Tumor site				
Intrahepatic	94	1		
Extrahepatic	52	0.704	0.485–1.022	0.065

Multivariate analysis by Cox proportion hazard regression. HR, indicates hazard ratio. 95% CI, refers to the 95% confidence interval. *p-values less than 0.05 were considered as a statistical significance.

### Correlation between IL-6R expression and gemcitabine response in cholangiocarcinoma

To determine the correlation between IL-6R expression and the gemcitabine response in CCA patients, we performed IHC staining in CCA samples in which tumor tissues were treated with gemcitabine by histoculture drug response assay (HDRA) as previously described (Suksawat et al., 2019). Tumor tissues were treated with gemcitabine at concentrations of 1,000 and 1,500 μg/ml. After the experiments, CCA patients were evaluated according to their response to classify the tumor tissues of patients into response and non-response. The 40 CCA samples were divided into 3 groups including the patients who were responders (*n* = 5) and non-responders (*n* = 27) in low and high concentrations of gemcitabine and who had different responses in both doses (*n* = 8). Fisher’s exact test demonstrated a significant correlation of IL-6R with high expression and patients with non-response to gemcitabine (*p* = 0.014) (Table 3). IHC scores for IL-6R protein and HDRA results were analyzed for their correlation using bivariate analysis. The result revealed that IL-6R expression was positively correlated with patients with non-response to gemcitabine (r = 0.444; *p* = 0.004) (Table 4).

**TABLE 3:** Correlation of IL-6R expression with clinicopathological characteristics of CCA patients with gemcitabine response from HDRA results.

Factors	No. of patients (*n* = 40)	IL-6R expression		
		Low	High	*p*-value
Gemcitabine				
Response or non-response	8	7	1	
Response	5	2	3	
Non-response	27	8	19	0.014*
Sex				
Female	17	8	9	
Male	23	9	14	0.429
Age				
<61	16	7	9	
≥61	17	8	9	
N/A	7	2	5	0.701
Histological types				
Papillary	12	7	5	
Non-papillary	19	7	12	
N/A	9	3	6	0.409
Tumor site				
Intrahepatic	20	9	11	
Extrahepatic	11	5	6	
N/A	9	3	6	0.819
Tumor morphology				
Mass forming	7	3	4	
Intraductal growth	5	2	3	
Mixed	19	9	10	
N/A	9	3	6	0.917

Correlation between IHC, staining score of IL-6R, and clinicopathological features by Fisher’s exact test. *p-values less than 0.05 were considered as a statistical significance.

**TABLE 4:** Pearson correlation coefficients between IHC scores of IL-6R expression and gemcitabine response from HDRA result.

	CCA patients with non-response to gemcitabine
Pearson Correlation	0.444
*p*-value	0.004*
No. of patients	40

* *p*-values less than 0.05 were considered as a statistical significance.

## Discussion

Adjuvant chemotherapy after surgical treatment is crucial for improving the survival of patients with CCA. The difficulty is the patient’s poor response to available chemotherapy due to the high heterogeneity of CCA at the molecular level reducing or blocking the efficacy of therapies. Understanding the mechanism of the chemoresponse of this cancer might help to improve patients’ outcomes (Marin et al., 2018; Banales et al., 2020). CAFs are prominent cells in TME that can stimulate matrix stiffening through remodeling of the ECM, secretion of cytokines, chemokines, and growth factors, facilitating a state of chemoresistance (Hogdall et al., 2018). IL-6 is an inflammatory molecule which is produced and secreted by various cell types that confer an aggressive behavior and poor response to therapies in many cancers as well as CCA (Frampton et al., 2012; Kumari et al., 2016; Thongchot et al., 2018; Thongchot et al., 2021). The study from Sripa et al. revealed that the concentration of plasma IL-6 is significantly higher in CCA patients than healthy groups (Sripa et al., 2012). Moreover, CCA-derived CAFs can induce cell growth and migration ability of CCA cells *via* IL-6 secretion (Thongchot et al., 2018). Taken together, CAFs in CCA environment would be one of the TME that involved in IL-6 production and contribute to CCA development and progression. Consistently, in this study, we revealed the effect of CCA-derived CAFs on the gemcitabine resistance of CCA cells. CAFs were successfully isolated from CCA tissues and were selected upon their potential extracellular IL-6 secretion. CCA cells treated with rhIL-6 sustained their viability and increased resistance to gemcitabine treatment. Additionally, CCA-derived CAFs induced CCA cell viability and resistance to gemcitabine by activation of pSTAT3 and the proteins involved in cell viability, as well as inducing IL-6 secretion in CCA cells. These results suggested that the IL-6 secreted from CAF plays roles in the gemcitabine resistance of CCA cells mediated by the IL-6/STAT3 signaling pathway. Supporting this finding, the efflux of the gemcitabine and conversion to gemcitabine monophosphate effects were examined. We found that these mechanisms were not involved in CAF-induced CCA gemcitabine resistance (Supplementary Figure S1). Furthermore, a previous study found that the conditioned medium of CCA-derived CAFs containing IL-6 stimulated the secretion of IL-6 (Thongchot et al., 2018). The IL-6-mediated STAT3 activation has been reported to cause therapeutic resistance in tumors by inducing several pro-survival pathways. The literature on the IL-6 functions on cancers reports that IL-6 binds to its receptor (IL-6R) and forms a complex with glycoprotein 130 (IL-6Rβ) to activate the downstream protein kinases, and subsequently activates STAT1, 3, and 5 (Heinrich et al., 2003; Babon et al., 2014; Jones and Jenkins, 2018) contributing to promotion of malignancy in colon cancer and hepatocellular carcinoma (HCC) (Ahmad et al., 2017; Sun et al., 2018). Additionally, IL-6 acts as a stromal driver of therapeutic resistance by activating epithelial-to-mesenchymal transition in esophageal adenocarcinoma (EAC) which enhances the treatment. Likewise, inhibition of IL-6 restored drug sensitivity in the patient-derived organoid culture of EAC cells (Ebbing et al., 2019). IL-6-induced STAT3 phosphorylation in pancreatic ductal adenocarcinoma (PDAC) was suppressed by blocking IL-6R leading to the attenuation of STAT3 activation in TME toward enhanced sensitivity of cancer cells to chemotherapy (Long et al., 2017). In colorectal cancer, IL-6 promoted cell proliferation and drug resistance through its downstream signaling molecules, such as STAT3, representing potential molecular targets for cancer therapy (Ying et al., 2015). Therefore, inhibition of CAF-CCA interaction could be considered as a potent therapeutic approach for cancers associated with IL-6/STAT3 activation (Masjedi et al., 2018). Blockade of IL-6R with a humanized monoclonal antibody (Tocilizumab; TCZ) is sufficient to inhibit Bmi-1 expression and overcome the intrinsic chemoresistance of head and neck cancer stem cells (Herzog et al., 2021). Hence, again, targeting the IL-6/STAT3 axis might be a promising outcome in cancers.

TCZ, anti-IL-6R mAbs, was used in this study to inhibit the ligation of IL-6/IL-6R in CAFs and CCA cells. We found that TCZ suppressed pSTAT3 levels in rhIL-6-induced CCA cells. A combination of TCZ and gemcitabine can enhance the sensitivity of CAF-induced gemcitabine resistance in KKU-213A cells by suppressing of pSTAT3 levels and Cyclin D1, with decreased levels of IL-6 secretion in the co-culture conditions. Consistent with non-small cell lung cancer (NSCLC) cells, TCZ decreased cell proliferation and induced the accumulation of sub-G1 phase in the cell cycle as well as possibly activating the NFκB pathway (Kim et al., 2015). However, TCZ could not affect cell viability and molecular changes in KKU-055 cells. To prove this finding, we focused on the expression level of IL-6R in both CCA cell lines and its correlation with the clinicopathological features of CCA patients. We found that KKU-055 displayed a lower level of IL-6R expression than KKU-213A. In addition, CCA patients who had a high expression of IL-6R without receiving any chemotherapy had the shortest overall survival rate when compared to other patient groups. Moreover, IL-6R can be used as an independent prognostic factor for the clinicopathological characteristics used to predict the overall survival rate of CCA patients. In colon cancer, in which IL-6R was correlated with the tumor size of the patients, a role of IL-6R in colorectal cancer progression was suggested and its possible roles as a biomarker could be useful in the follow-up disease and as potential targets for the therapy (Waldner et al., 2012; Turano et al., 2021). In epithelial ovarian cancer (EOC), the expression of IL-6 and IL-6R was increased in therapy-resistant cells and correlated with chemoresistance of EOC cells. The authors suggested that blockade of the IL-6 signaling pathways might reduce production and secretion of IL-6 leading to an increase in the potential effect of the agent involving inhibition of IL-6 or IL-6R in cancer (Yousefi et al., 2019). In breast cancer, high levels of soluble IL-6R (sIL-6R) in a patient’s sera are likely to be associated with recurrence-free survival when compared to those patients with low levels of sIL-6R (Won et al., 2013). High expression of IL-6R was also observed and indicated a poor prognosis for overall survival and metastasis-free survival in patients with soft tissue sarcomas (Nakamura et al., 2020).

Subsequently, we analyzed the correlation between IL-6R expression and gemcitabine response from the HDRA results to confirm that the IL-6R expression level was correlated with the gemcitabine response in CCA patients. The results showed a positive correlation of IL-6R with a high expression in the patients who were non-responders to gemcitabine. These findings confirmed that CCA with a high level of IL-6R has a poor response to gemcitabine treatment, which is consistent with cell studies. As mentioned, KKU-213A cells with a high IL-6R expression level indicate a high gemcitabine resistance ability rather than KKU-055 cells and the combination with the IL-6R inhibitor can enhance gemcitabine sensitivity in KKU-213A cells. In contrast to CCA with a low level of IL-6R, gemcitabine strongly affects CCA cells (KKU-055) and the combination with IL-6R inhibitor is not necessary. The IL-6R expression, therefore, is useful as a predictive marker for a personalized therapy in CCA patients.

**FIGURE 8:**
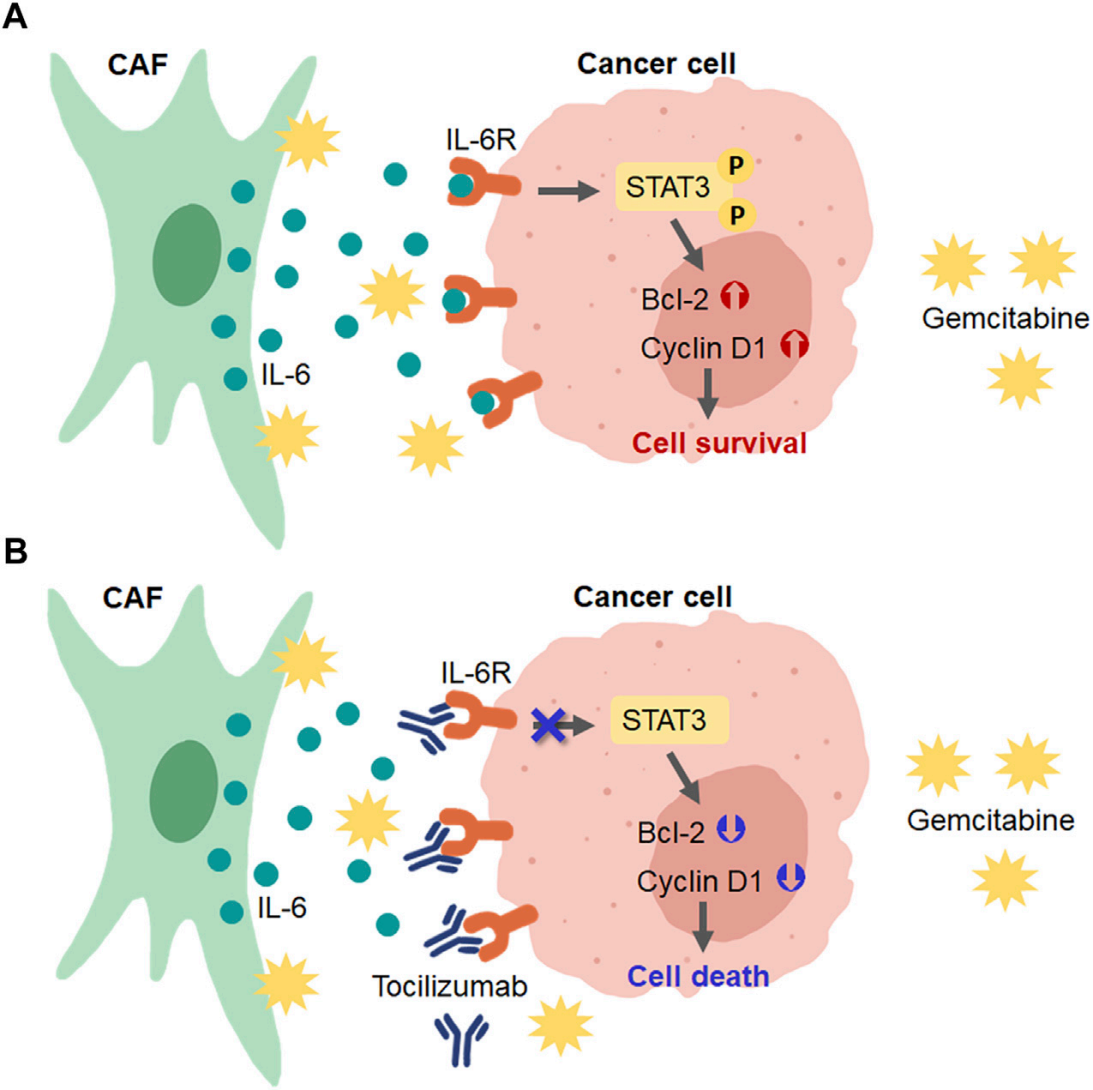
Schematic diagram for the role of CAFs in CCA cell resistance to gemcitabine and the presence of IL-6R blockage by tocilizumab. **(A)** CAFs secreted IL-6 to induce IL-6R/STAT3 signaling and upregulated the expression of proteins involved in the cell survival mechanism in CCA cells. **(B)** Blocking the IL-6-IL-6R binding in CCA cells by tocilizumab inhibited CAF-CCA interaction overcame the gemcitabine resistance and induced CCA cell death.

In conclusion, our findings show that CCA-derived CAFs induced gemcitabine resistance in CCA cells *via* the activation of IL-6/STAT3 signaling. IL-6R can be a prognostic factor for the overall survival rate of CCA patients. IL6R-blocking antibody can induce chemosensitivity by attenuating the STAT3 activation in high IL-6R expressing CCA cells (Figure 8). Therefore, the IL-6/STAT3 axis could be a potential targeting pathway for CCA treatment. IL-6R expression, therefore, is useful as a predictive marker for a personalized therapy in CCA patients.

## Data Availability

The original contributions presented in the study are included in the article/Supplementary Material, further inquiries can be directed to the corresponding author.
