# Supplementary material for: Interleukin-6-derived cancer-associated fibroblasts activate STAT3 pathway contributing to gemcitabine resistance in cholangiocarcinoma
Source: Front Pharmacol. 2022 Aug 26;13:897368. doi: 10.3389/fphar.2022.897368 (PMC9459012; doi:10.3389/fphar.2022.897368)
Supplement: Supplementary file 1 [file Table1.pdf]

Supplementary Material

Table S1. The clinical information of isolated CAFs from 8 CCA patients

CAFs	Sex	Age	Tumor site	Tumor morphology	Margin	Histology	Lymph node status	TNM Stage			Stage
								T	N	M	
CAF1	M	50	Perihilar	MF+PI	R0	Tubular	N1	3	1	1	IVB
CAF2	M	65	Hepatectomy not done								
CAF3	M	61	N/A	MF	N/A						
CAF4	M	83	Intrahepatic bile duct	MF+PI	R0	Tubular	N2	4	1	0	IVA
CAF5	M	65	Intrahepatic bile duct	MF+PI	R1	Adeno (well diff)	N0	4	0	x	IVA
CAF6	M	65	Perihilar	MF+PI	R2	Adeno (well diff)	N0	2a	0	x	II
CAF7	M	60	Perihilar	MF+ID	R0	Papillary invasive	N0	3	0	x	IIIB
CAF8	F	58	Intrahepatic bile duct	MF	R1	Adeno (well diff)	Nx	3	x	x	III

MF; mass forming, PI; periductal infiltrating; ID; intraductal.
