# Supplementary figures and images for: Interleukin-6-derived cancer-associated fibroblasts activate STAT3 pathway contributing to gemcitabine resistance in cholangiocarcinoma
Source: Front Pharmacol. 2022 Aug 26;13:897368. doi: 10.3389/fphar.2022.897368 (PMC9459012; doi:10.3389/fphar.2022.897368)

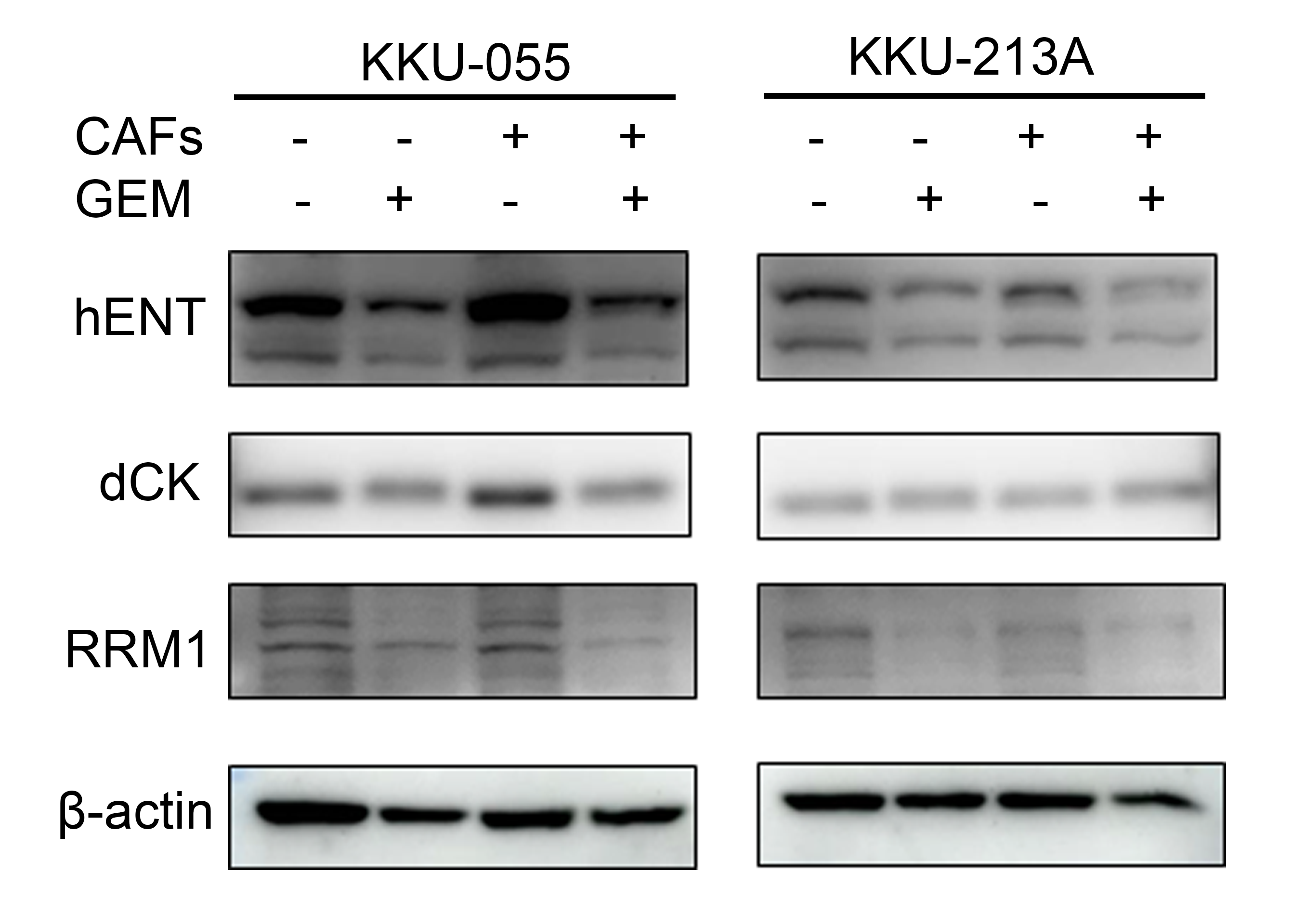

Supplement: Supplementary file 2 [file Image1.tif]
